# Research on the establishment of NDVI long-term data set based on a novel method

**DOI:** 10.1038/s41598-023-36939-y

**Published:** 2023-06-17

**Authors:** Dongliang Fan

**Affiliations:** China Meteorological Administration Training Center, Beijing, 100081 People’s Republic of China

**Keywords:** Climate sciences, Ecology

## Abstract

This study compares the relationship between different NDVI (Normalized Difference Vegetation Index), the NDVI of AVHRR (Advanced Very High Resolution Radiometer) (NDVIa), the NDVI of MODIS (Moderate Resolution Imaging Spectrometer) (NDVIm), and the NDVI of VIRR (Visible and Infrared Radiometer) (NDVIv), and found that there is a significant correlation between the NDVIa and the NDVIm, and between the NDVIv and the NDVIa, the relationship between the three is NDVIv < NDVIa < NDVIm. Machine learning is an important method in artificial intelligence. It can solve some complex problems through algorithms. This research uses linear regression algorithm in machine learning to construct the Fengyun Satellite NDVI correction method. By constructing a linear regression model, the NDVI value of Fengyun Satellite VIRR is corrected to a level that is basically the same as NDVIm. The corrected correlation coefficients (R^2^) were significantly improved, and the corrected correlation coefficients were significantly improved, and the confidence levels were all significant correlations less than 0.01. It is proved that the corrected normalized vegetation index of Fengyun Satellite has significantly improved accuracy and product quality compared with the normalized vegetation index of MODIS.

## Introduction

Fengyun-3 (FY-3) is China's second-generation polar orbiting meteorological satellite. It can obtain global, all-weather, three-dimensional, quantitative, multi-spectral atmospheric, land surface and sea surface characteristic parameters. FY-3A and FY-3B both carry VIRR. The VIRR has 10 spectral bands (0.43–12.50 μm) and the spatial resolution is 1.1 km. It is mainly used to monitor the global cloud cover, ocean surface temperature, and vegetation growth status and types^1^. Terra and Aqua are satellites in the US (United States) Earth Observation System (EOS) program. Both satellites are equipped with MODIS. MODIS has 36 bands (0.405–14.385 μm), among which the spatial resolution of band 1–2 is 250 m, and the spatial resolution band 3–7 is 500 m, the spatial resolution of band 8–36 is 1000 m, and the scan width is 2330 km. It can be used for long-term global observation of the earth's surface, biosphere, solid earth, atmosphere and ocean. MODIS has stable performance and good calibration, and has obtained many years of data since its launch^2–4^. If the data of Terra/MODIS and Aqua/MODIS are compared with FY-3/VIRR, the performance of FY-3 can be preliminary evaluated, which can supplement and correct to a certain extent. AVHRR is the main detection instrument of the National Oceanic and Atmospheric Administration (NOAA) series of satellites. It is a scanning radiometer with five spectral channels. The scanning angle of the on-board detector is ± 55.4°, which is equivalent to detecting a band of 2800 km wide on the ground. The sub-satellite point resolution of AVHRR is 1.1 km. There are currently two types of AVHRR data on a global scale: NOAA Global Area Coverage (GAC) data and NOAA Global Vegetation Index (GVI) data^5^. GVI is composed of pixels with the largest NDVI value in images for 7 consecutive days. AVHRR has been producing global vegetation data since 1982^6–8^.

Vegetation plays an extremely important role in the earth's ecosystem. It affects the climate, hydrology and biochemical environment while being restricted by these factors. Therefore, vegetation is an important indicator to measure the impact of climate, humanities and biochemical factors on the environment^9–12^. NDVI is a commonly used vegetation index. Because of its simple calculation, wide spatial coverage, and high detection sensitivity, it is widely used in vegetation monitoring and is one of the important parameters describing the characteristics of surface vegetation^13,14^. At present, a large number of scholars have conducted interactive comparisons between different sensors^15,16^. For example, Duarte et al.^12^ derived phenological indexes from NDVI through an open source tool developed by QGIS (A free and open source geographic information system). In the present work, the developed toolbar was applied to MODIS data covering a particular region of Portugal, which can be generally applied to other satellite data and study area. The code is open and can be modified according to the user requirements. Other advantage in publishing the plug-ins and the application code is the possibility of other users to improve this application. Feng Rui et al.^17^ conducted a differential analysis on the NDVI of FY/MERSI (Medium Resolution Spectral Imager) and EOS/MODIS and found that the inversion results of various ground features showed good linear consistency; Yuan Zhengwu et al.^18^ established a quantitative relationship between Landsat TM (Thematic Mapper) and HJCCD (Environmental satellite CCD camera) vegetation index, which provides a basis for the comprehensive application of Landsat TM and HJCCD data; Xu Hanqiu et al.^19^ analyzed the characteristics of the red band and near-infrared band of ASTER (Advanced Spaceborne Thermal Emission and Reflection Radiometer) and Landsat ETM + (Enhanced Thematic Mapper) by comparing the vegetation index. Wu Wenbin et al.^20^ compared the Savitzky-Golay filter method for fitting NDVI time series data and the asymmetric Gaussian function fitting method for NDVI time series data; Li Jing et al.^21^ used the NDVI data of the Southwest Virginia coal field in the United States from 1984 to 2010 as the data source, and compared the filtering algorithms of the three long-term remote sensing vegetation index data sets of TIMESAT3.1 (Time-series Satellite data Analysis Tool 3.1); Sha Sha et al.^22^ took Maqu as an example to compare and analyze the three sets of NDVI long-term series indices, NDVI/MODIS, NDVI/GIMMS (Global Inventory Modeling and Mapping Studies) and NDVI/NSMC (National Center for Space Weather). The vegetation index of a longer time series is a simple and effective dynamic monitoring research parameter, which is very important for monitoring surface vegetation, ecological improvement, ecological evaluation and so on^12,17–22^.

Machine learning is an important method in artificial intelligence. It can solve some complex problems through algorithms and has become one of the most popular subjects at the moment^23–26^. The application of machine learning in remote sensing is generally divided into the following steps^27,28^: collecting and cleaning data, model building, selecting the correct algorithm, obtaining reliable results, and visualizing data. In remote sensing technology, people mainly use satellites or drones to collect data^29^. Data cleaning occurs when our data set is incomplete or missing values, and the choice of algorithm involves understanding one of the problems to be solved. If the model is only for forecasting, not for obtaining high-reliability results, then this workflow will end here. However, if a person is writing a research paper, or wants to obtain highly credible results, then you need to use a graphics library to plot the results and get the true solution from the chart data^30^.

A training sample set was built based on linear regression algorithm, combining the normalized vegetation index products retrieved by Fengyun satellite and MODIS and observation parameters, surface type, ground elevation and meteorological factors. Therefore, the NDVI product retrieved by Fengyun Satellite is corrected to the NDVI product that is basically consistent with MODIS through the machine learning model, related factors and related parameters, and a long-term normalized vegetation index is obtained.

Because of the difference of sensitivity, resolution and observation method, different detection instruments have certain differences in the detection value of NDVI. Therefore, this study compares the NDVI of MODIS with the NDVI of VIRR and AVHRR respectively. We use statistical methods to compare and analyze the normalized vegetation index of the three, and find the difference and correlation between the normalized vegetation index of VIRR, AVHRR and MODIS; based on the machine learning algorithm, the Fengyun Satellite NDVI correction algorithm is constructed in order to form a long time series of vegetation index.

## Data and methods

### Data

This paper selects parts of China and surrounding areas as the research area. The research data selects the NDVI data of MODIS (NDVIm) and AVHRR (NDVIa) sensors on Terra and Aqua, and the NDVI data of VIRR (NDVIv) sensors on Fengyun satellite^31^. (I) Compare the NDVIv with the NDVIa, and the NDVIa and NDVIm. (II) Find out the functional relationship between NDVIa and NDVIm, and the functional relationship between NDVIv and NDVIa through comparison. (III) use NDVIa to correct NDVIv data to a level equivalent to NDVIm.

The data used in this study include (see Table 1): NDVIa from 1982 to 2015, NDVIm from 2000 to 2019, and NDVIv from 2015 to 2020, all of which have a resolution of 0.05°. Because in 2005, there are both NDVIa data and NDVIm data. Therefore, we use the data of this year to compare NDVIa and NDVIm, and explore the correlation between the two. Because in 2015, there are both NDVIv data and NDVIa data. Therefore, we used the data of this year to compare NDVIv and NDVIa and explore the correlation between the two. Finally, we compared the corrected NDVIv of 2019 with the NDVIm of 2019 to verify the success of the model we constructed.

**Table 1:** Study data information.

Serial numbers	Data sources	Data dates (year)	Data resolution (degree)
1	NDVIa	1982–2015	0.05
2	NDVIm	2000–2019	0.05
3	NDVIv	2015–2020	0.05

**Figure 1:**
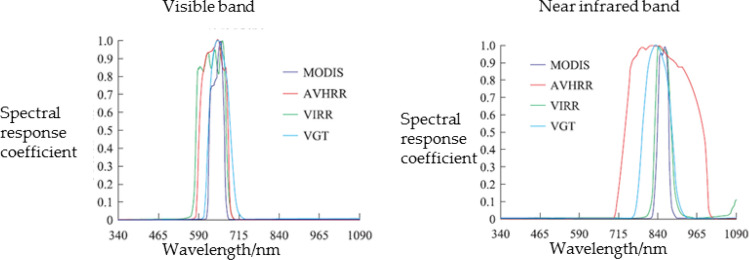
Spectral response function curves of different satellite sensors in the visible and near-infrared spectrum^29^.

Figure 1 shows the spectral response function curves of different satellite sensors in the visible and near-infrared spectrum^32^. By comparison, it can be found that in the visible light band, the spectral response function of MODIS is narrower than AVHRR, and the spectral response function of AVHRR is narrower than VIRR. In the near-infrared band, MODIS still has the narrowest spectral response function, followed by VIRR, and AVHRR has the widest spectral response function. The channel, wavelength range, corresponding spectrum and sub-satellite resolution information of MODIS, AVHRR, and VIRR sensors are shown in Table 2.

**Table 2:** Corresponding channels of the main NDVI data source sensors.

Band	Aisle	Wavelength range/nm	Corresponding spectrum	Resolution of sub-satellite point/m
Visible band	MODIS-1	620–670	Red	250
	AVHRR-1	580–680	Green–red	1100
	VIRR-1	580–680	Green–red	1100
Near infrared band	MODIS-2	841–876	Near infrared	250
	AVHRR-2	725–1000	Near infrared	1100
	VIRR-2	840–890	Near infrared	1100

### Method

**Figure 2:**
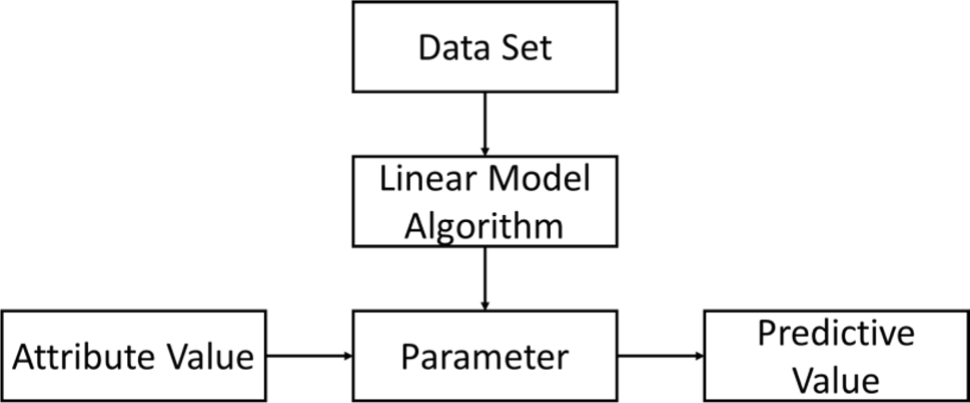
Schematic diagram of the linear regression algorithm flow.

Linear model is a form of machine learning model. The form of linear model is relatively simple and easy to model. The linear model contains some important basic ideas in machine learning. Many more powerful nonlinear models can be obtained by introducing hierarchical structure or high-dimensional mapping on the basis of linear models. There are many forms of linear models, and linear regression is a common one. Linear regression tries to learn a linear model to predict the real-valued output markers as accurately as possible. By establishing a linear model on the data set, a loss function is established, and finally the model parameters are determined with the goal of optimizing the cost function, so as to obtain the model for subsequent prediction. The general linear regression algorithm process is as presented in Fig. 2.

The detailed procedure is as follows^33^:

(I) The data is standardized and preprocessed. The preprocessing includes data cleaning, screening, organization, etc., so that the data can be input into the machine learning model as feature variables.
(II) Different machine learning algorithms are selected to train a separate data set, and find the best machine learning model, establish a machine learning model based on the normalized vegetation index product retrieved by Fengyun satellite.
(III) Verify and output the long-term series normalized vegetation index of the Fengyun satellite.

For 2001–2005, there are both AVHRR NDVI data and MODIS NDVI data. Therefore, we used the data of these 5 years to compare NDVIa and NDVIm and explore the correlation between the two. Because 2015 has both VIRR's NDVI data and AVHRR's NDVI data. Therefore, we used the data of this year to compare NDVIv and NDVIa and explore the correlation between the two. Finally, we compared the corrected NDVIv of 2019 with the NDVIm of 2019 to verify the success of the model we constructed.

The linear machine learning model is used to construct the optimal functional relationship between the NDVIa and the NDVIm. The formula is as presented in formula (1):

1 $${\text{Y}}_{{{\text{NDVIm}}}} = \left\{ {{\text{k2}}00{1},{\text{k2}}00{2},{\text{k2}}00{3},{\text{k2}}00{4},{\text{k2}}00{5},{\text{kmin}},{\text{kmax}},{\text{kave}}} \right\} \times {\text{X}}_{{{\text{NDVIa}}}} + \left\{ {{\text{m2}}00{1},{\text{m2}}00{2},{\text{m2}}00{3},{\text{m2}}00{4},{\text{m2}}00{5},{\text{mmin}},{\text{mmax}},{\text{mmean}}} \right\}$$

In the formula, X_NDVIa_ is the NDVI value of AVHRR, Y_NDVIm_ is the NDVI value of MODIS, k is the coefficient value of the linear function relationship between NDVIa and NDVIm, k2001, k2002, k2003, k2004, k2005, kmin, kmax, kave are the coefficients of 2001, 2002, 2003, 2004, 2005, the 5-year minimum, 5-year maximum, and the 5-year coefficient average respectively. m is the intercept of the linear function relationship between the NDVIa and the NDVIm, m2001, m2002, m2003, m2004, m2005, mmin, mmax, mmean are the intercept of 2001, 2002, 2003, 2004, 2005 Year, 5-year minimum, 55-year maximum, and 5-year average respectively.

Through multiple cross-comparison analysis, the optimal coefficient k and the optimal coefficient m are selected, and then the optimal functional relationship between NDVIa and NDVIm is determined.

Based on the above analysis, we continue to construct the functional relationship between NDVIa and NDVIv, according to formula (2).

2 $${\text{X}}_{{{\text{NDVIa}}}} = {\text{aZ}}_{{{\text{NDVIv}}}} + {\text{b}}{.}$$

In the formula (2), Z_NDVIv_ is the NDVI value of VIRR, X_NDVIa_ is the NDVI value of AVHRR, a is the coefficient value of the linear function relationship between the NDVIv and the NDVIa fitting, and b is the intercept of the linear function relationship between NDVIv and NDVIa fitting.

Replacing the functional relationship between NDVIa and NDVIv into the optimal NDVIa and NDVIm functional relationships filtered out to obtain the refitted NDVIv, which is Yvir_ndvi in the formula (3). The functional relationship formula of the simulated NDVIv is as follows (3):

3 $${\text{C}}_{{{\text{NDVIcv}}}} = {\text{k}}_{{{\text{NDVIa}}}} + {\text{m}} = {\text{k}}\left( {{\text{aZ}}_{{{\text{NDVIv}}}} + {\text{b}}} \right) + {\text{m}} = {\text{kaZ}}_{{{\text{NDVIv}}}} + {\text{kb}} + {\text{m}}{.}$$

In the formula, C_NDVIcv_ is the corrected NDVIv(NDVIcv), k is the optimal coefficient of the correlation between NDVIa and NDVIm, and m is the optimal intercept of the correlation between NDVIa and NDVIm.

The data of 2005 were selected to compare NDVIm and NDVIa in some parts of China and surrounding areas. The data of 2015 were selected to compare NDVIv and NDVIa in some parts of China and surrounding areas. Through analysis, the correlation among NDVIv, NDVIa and NDVIm is found.

## Results

### Comparison of NDVI between AVHRR and MODIS in parts of China and surrounding areas

**Figure 3:**
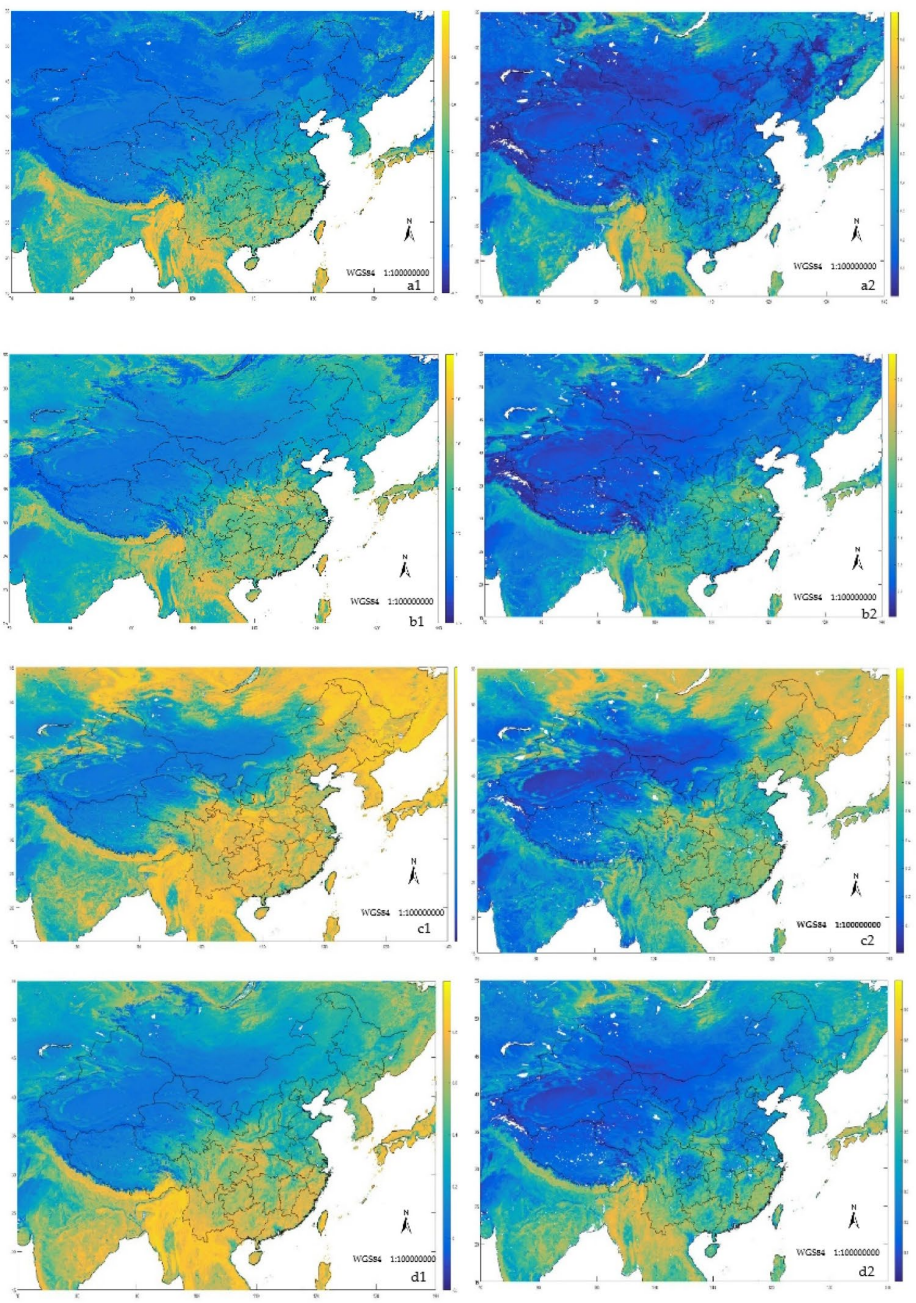
NDVI values monitored by AVHRR and MODIS in parts of China and surrounding areas in January, April, July and October 2005 (a1 is the NDVIa of January, a2 is the NDVIm of January; b1 is the NDVIa of April, b2 is the NDVIm of April;c1 is the NDVIa of July, c2 is the NDVIm of July;d1 is the NDVIa of October, d2 is the NDVIm of October) (Made by: Python 3.8.3 https://www.python.org/downloads/release/python-383/).

By comparing the NDVIa and NDVIm in parts of China and surrounding areas (Table 3), we found that the correlation coefficient for January, April, July, and October of 2005 was between 0.8652 and 0.9348, and the coefficient of determination was between 0.7024 and 0.8519. The confidence p is at the level of 0.01 or 0.05, indicating that the NDVIa and the NDVIm have a good correlation. Through comparison, it can be found that overall, in comparison with the NDVIa and NDVIm in 2005, except that the NDVIa in January was larger than the NDVIm, the NDVIa in the remaining months were all smaller than the NDVIm. As can be seen from the Fig. 3, in the comparison between April and October, the area with lower NDVI value, the NDVIa is greater than the NDVIm.

**Table 3:** Comparison results of NDVIa and NDVIm.

Time	Fitting function	Correlation coefficient (r)	Decisive factor (R2)	Confidence (p)	AVHRR/NDVI compared with MODIS/NDVI
January	Y_NDVIm_ = 1.0679 × X_NDVIa_ − 0.0564	0.8652	0.7024	0.0175 < 0.05	NDVIa > NDVIm
April	Y_NDVIm_ = 1.1594 × X_NDVIa_ − 0.0304	0.9115	0.8097	0.0081 < 0.01	NDVIm > NDVIa
July	Y_NDVIm_ = 1.0949 × X_NDVIa_ + 0.0364	0.9348	0.7864	0.0166 < 0.05	NDVIm > NDVIa
October	Y_NDVIm_ = 1.1870 × X_NDVIa_ − 0.0267	0.8996	0.8519	0.0090 < 0.01	NDVIm > NDVIa

*Is the confidence level of 0.05, ** is the confidence level of 0.01.

### Comparison of NDVI between VIRR and AVHRR in parts of China and surrounding areas

**Figure 4:**
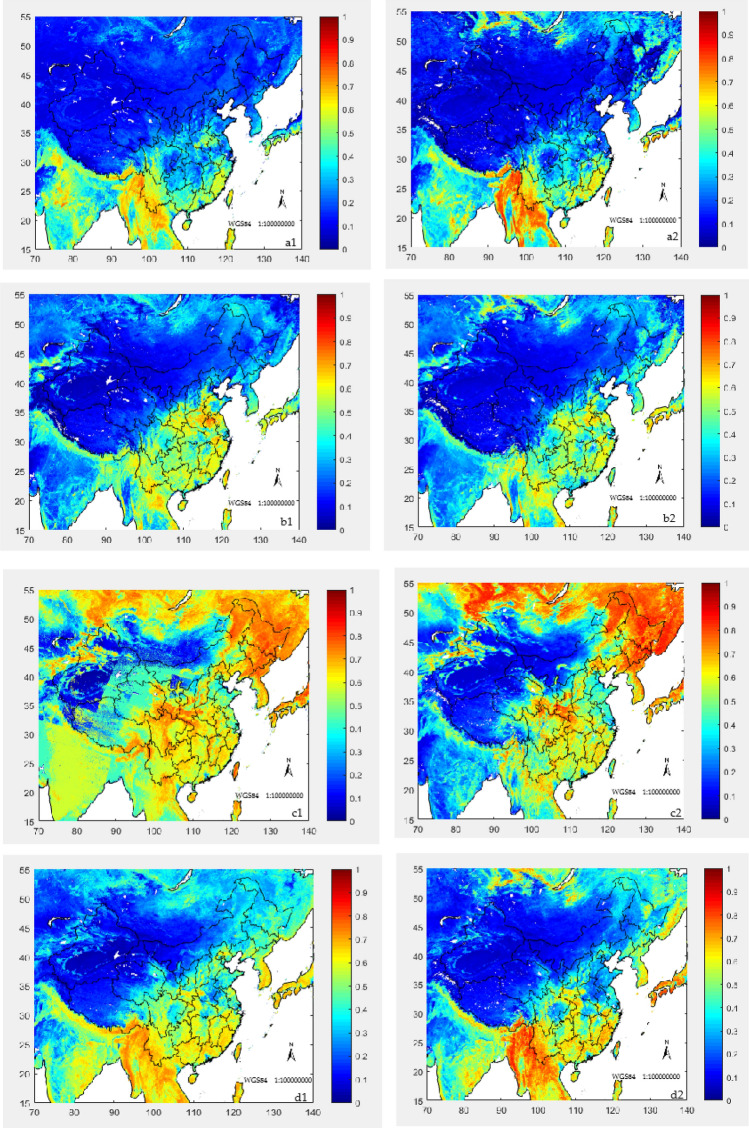
The NDVI values monitored by VIRR and AVHRR in parts of China and surrounding areas in January, April, July and October 2015 (a1 is the NDVIv of January, a2 is the NDVIa of January; b1 is the NDVIv of April, b2 is the NDVIv of April; c1 is the NDVIv of July, c2 is the NDVIv of July; d1 is the NDVIv of October, d2 is the NDVIa of October) (Made by: Python 3.8.3 https://www.python.org/downloads/release/python-383/).

By comparing the NDVIv and NDVIa in parts of China and surrounding areas (Table 4), we found that the correlation coefficient for January, April, July, and October of 2015 was between 0.7238 and 0.8929, and the coefficient of determination was between 0.6072 and 0.8299. The confidence p is at the level of 0.01 or 0.05, indicating that there is a significant correlation between the NDVIv and the NDVIa. The NDVIa can be used to correct the NDVIv. Through comparison, it can be found that, on the whole, the NDVIv in January, April, July, and October of 2015 is smaller than the NDVIa. However, it can be seen from Fig. 4 that in the comparison in April, the NDVIv is greater than the NDVIa in areas with lower NDVI values.

**Table 4:** Comparison results of NDVIv and NDVIa in 2015.

Time	Fitting function	Correlation coefficient (r)	Decisive factor (R2)	Confidence (p)	AVHRR/NDVI compared with VIRR/NDVI
January	X_NDVIa_ = 0.9934 × Z_NDVIv_	0.8690	0.7585	0.0097 < 0.01	NDVIa > NDVIv
April	X_NDVIa_ = 0.8591 × Z_NDVIv_ + 0.0402	0.8929	0.7693	0.0067 < 0.01	NDVIv > NDVIa (in low-value area); NDVIa > NDVIv (in other areas)
July	X_NDVIa_ = 0.9602 × Z_NDVIv_ − 0.0509	0.7238	0.6072	0.0202 < 0.05	NDVIa > NDVIv
October	X_NDVIa_ = 0.9888 × Z_NDVIv_ − 0.0153	0.8698	0.8299	0.0073 < 0.01	NDVIa > NDVIv

*Is the confidence level of 0.05, ** is the confidence level of 0.01.

Through the comparative analysis of 3.1 and 3.2, we found that there is a significant correlation between the NDVIa and the NDVIm, and the NDVIa is smaller than the NDVIm, and there is also a significant relationship between the NDVIv and the NDVIa. and the NDVIv is smaller than the NDVIa. Therefore, we deduce the following correlation: NDVIv < NDVIa < NDVIm.

### Construction of NDVI correction algorithm based on linear machine learning model

We construct a linear machine learning model according to the 2.2 method.

In order to increase our selectivity in the linear model and the accuracy of the correction, we respectively compared the NDVIa and the NDVIm from January, April, July, and October from 2001 to 2005. The specific analysis and comparison results are as follows.

By comparing the NDVIa and the NDVIm in January (Table 5), it is found that from 2001 to 2005, the coefficients of the NDVIa and the NDVIm were 1.0358–1.0679, the average coefficient was 1.0553, and the intercept was − 0.0564 to − 0.0285. The average intercept is − 0.0409, the correlation coefficient r is 0.8638–0.8768, the average correlation coefficient is 0.8693, the determination coefficient R^2^ is 0.7024–0.7662, the average correlation coefficient is 0.7400, and the confidence is 0.0133–0.0175, all of which are at the 0.05 level of confidence. The average confidence is 0.0149.

**Table 5:** Comparison and analysis of NDVIa and NDVIm in January from 2001 to 2005.

Year	Coefficient	Tntercept	Correlation coefficient (r)	Decisive factor (R^2^)	Confidence(p)
2001	1.0504	− 0.0441	0.8689	0.7504	0.0144*
2002	1.0563	− 0.0353	0.8716	0.7570	0.0133*
2003	1.0663	− 0.0403	0.8768	0.7662	0.0137*
2004	1.0358	− 0.0285	0.8638	0.7239	0.0157*
2005	1.0679	− 0.0564	0.8652	0.7024	0.0175*
Average	1.0553	− 0.0409	0.8693	0.7400	0.0149*

*Is the confidence level of 0.05, ** is the confidence level of 0.01, *** is the confidence level of 0.001.

By comparing the NDVIa and the NDVIm in April (Table 6), it is found that from 2001 to 2005, the coefficients of the NDVIa and the NDVIm were 1.1504–1.1823, the average coefficient was 1.1637, and the intercept was − 0.0415 to − 0.0272. The average intercept is − 0.0332, the correlation coefficient r is 0.9070–0.9137, the average correlation coefficient is 0.9102, the determination coefficient R^2^ is 0.8070–0.8362, the average correlation coefficient is 0.8184, and the confidence is 0.0069–0.0081, which are all within the confidence level of 0.01. The average confidence is 0.0076.

**Table 6:** Comparison and analysis of NDVIa and NDVIm in April from 2001 to 2005.

Year	Coefficient	Tntercept	Correlation coefficient (r)	Decisive factor (R^2^)	Confidence (p)
2001	1.1643	− 0.0415	0.9080	0.8070	0.0078**
2002	1.1823	− 0.0369	0.9109	0.8205	0.0076**
2003	1.1619	− 0.0272	0.9137	0.8362	0.0069**
2004	1.1504	− 0.0298	0.9070	0.8185	0.0074**
2005	1.1594	− 0.0304	0.9115	0.8097	0.0081**
平均	1.1637	− 0.0332	0.9102	0.8184	0.0076**

*Is the confidence level of 0.05, **is the confidence level of 0.01, ***is the confidence level of 0.001.

Through the comparison of the NDVIa and the NDVIm in July (Table 7), it is found that from 2001 to 2005, the coefficients of the NDVIa and the NDVIm are 1.0928–1.1191, the average coefficient is 1.1026, the intercept is 0.0229–0.0382, and the average intercept is 1.0928–1.1191. The distance is 0.0301, the correlation coefficient r is 0.9341–0.9395, the average correlation coefficient is 0.9370, the determination coefficient R2 is 0.7741–0.8008, the average correlation coefficient is 0.7870, and the confidence is 0.0149–0.0173, all at the 0.05 level of confidence, the average confidence It is 0.0160.

**Table 7:** Comparison and analysis of NDVIa and NDVIm in July from 2001 to 2005.

Year	Coefficient	Tntercept	Correlation coefficient (r)	Decisive factor (R^2^)	Confidence (p)
2001	1.0928	0.0270	0.9372	0.7741	0.0173*
2002	1.1061	0.0229	0.9395	0.8008	0.0149*
2003	1.1191	0.0382	0.9341	0.7772	0.0157*
2004	1.0999	0.0258	0.9392	0.7964	0.0153*
2005	1.0949	0.0364	0.9348	0.7864	0.0166*
平均	1.1026	0.0301	0.9370	0.7870	0.0160

*Is the confidence level of 0.05, **is the confidence level of 0.01, ***is the confidence level of 0.001.

By comparing the NDVIa and NDVIm in October (Table 8), it is found that from 2001 to 2005, the coefficient of NDVIa and NDVIm was 1.1349–1.1809, the average coefficient was 1.1523, and the intercept was − 0.0330 to − 0.0113. The average intercept is -0.0189, the correlation coefficient r is 0.8903–0.9048, the average correlation coefficient is 0.8985, the determination coefficient R^2^ is 0.8521–0.8777, the average correlation coefficient is 0.8619, and the confidence is 0.0071–0.0088, which are all within the confidence level of 0.01. The average confidence is 0.0081.

**Table 8:** Comparison and analysis of NDVIa and NDVIm in October from 2001 to 2005.

Year	Coefficient	Tntercept	Correlation coefficient (r)	Decisive factor (R^2^)	Confidence (p)
2001	1.1433	− 0.0165	0.8977	0.8627	0.0080**
2002	1.1349	− 0.0171	0.8903	0.8777	0.0071**
2003	1.1547	− 0.0168	0.9048	0.8599	0.0082**
2004	1.1476	− 0.0113	0.8977	0.8571	0.0085**
2005	1.1809	− 0.0330	0.9019	0.8521	0.0088**
平均	1.1523	− 0.0189	0.8985	0.8619	0.0081**

*Is the confidence level of 0.05, **is the confidence level of 0.01, ***is the confidence level of 0.001.

### Comparative analysis of the revised NDVIv and NDVIm

**Figure 5:**
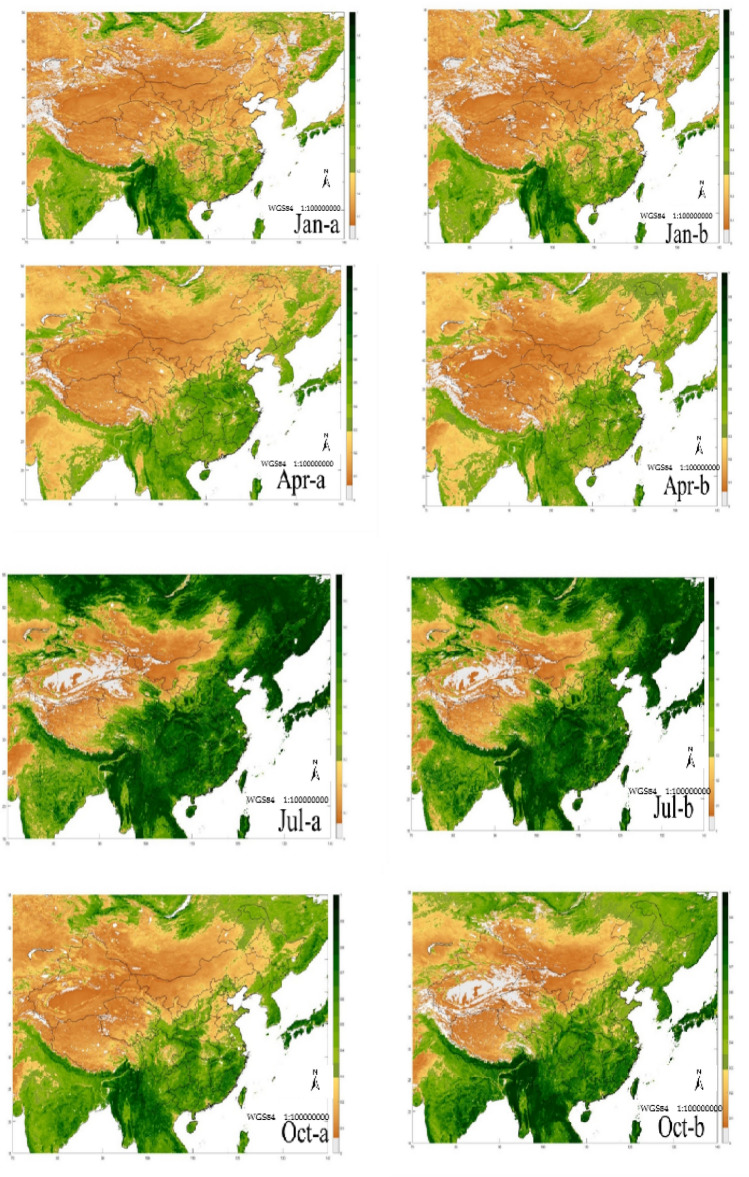
The revised NDVI value comparison between VIRR and MODIS in January, April, July, and October of 2019 in parts of China and surrounding areas (Jan-a is the revised NDVIv of January, Jan-b is the NDVIm of January; Apr-a is the revised NDVIv of April, Apr-b is the NDVIm of April; Jul-a is the revised NDVIv of July, Jul-b is the NDVIm of July; Oct-a is the revised NDVIv of October, and Oct-b is the NDVIm of October) (Made by: Python 3.8.3 https://www.python.org/downloads/release/python-383/).

Use the methods and data of 2.2 and 3.2 to construct a linear machine learning model, correct the NDVIv in parts of China and surrounding areas, find the best fitting function of the corrected NDVIv, and correct the NDVIv values in parts of China and surrounding areas in January, April, July, and October. Simultaneously compare the NDVIm in the same area. The constructed fitting model and comparative analysis are shown in Fig. 5 and Table 9.

**Table 9:** The optimal function construction of NDVIv fitting and its comparison with NDVIm.

Time	Optimal function of NDVIv fitting	Correlation coefficient (r) before correction	Corrected correlation coefficient®	Coefficient of determination (R^2^) before correction	Corrected coefficient of determination (R^2^)	Confidence (p)
January	C_NDVIcv_ = 1.0483Z_NDVIv_ − 0.04092	0.8690	0.9445	0.7585	0.9326	0.0092**
April	C_NDVIcv_ = 0.9997Z_NDVIv_ + 0.01572	0.8929	0.9345	0.7693	0.9193	0.0073**
July	C_NDVIcv_ = 1.0746Z_NDVIv_ − 0.09516	0.7238	0.9126	0.6072	0.9002	0.0003***
October	C_NDVIcv_ = 1.1394Z_NDVIv_ − 0.03407	0.8698	0.9329	0.8299	0.9233	0.0012**

*Is the confidence level of 0.05, **is the confidence level of 0.01, ***is the confidence level of 0.001.

After the NDVIv is corrected, compared with the NDVIm (Table 8), the correlation coefficient between the NDVIv and the NDVIm before correction is 0.7238–0.8929, and the correlation coefficient after correction is increased to 0.9126–0.9445, and the correlation coefficient of NDVIv before correction is 0.9126–0.9445. The coefficient of determination of the NDVIv value and the NDVIm is 0.6072–0.8299, and the corrected coefficient of determination is increased to 0.9002–0.9326, and the confidence level is also increased from the original 0.05 or 0.01 level to above the 0.01 level, even reaching the 0.001 level confidence level. At the same time, it can be seen from Fig. 5 that the revised NDVIv has a substantially improved consistency compared with the NDVIm. Prove that our correction method is feasible.

## Conclusion and discussion

Through research, we found that there is a significant correlation between the NDVIa and the NDVIm, and there is also a significant correlation between the NDVIv and the NDVIa. The relationship between the three is NDVIv < NDVIa < NDVIm.

Using the constructed linear model in machine learning, the NDVIv was corrected, and compared with the NDVIm, there is a good consistency. The correlation coefficient before correction is 0.7238–0.8929, the correlation coefficient after correction is significantly improved to 0.9126–0.9445; the determination coefficient before correction is 0.6072–0.8299, the correlation coefficient after correction is significantly improved to 0.9002–0.9326, and the confidence levels are all significant correlations less than 0.01. It is proved that the corrected NDVIv has significantly improved accuracy and product quality compared with the NDVIm.

In addition, we have the following thoughts. Firstly, in this study, we use linear machine learning models to correct the NDVIv in parts of China and surrounding areas. In some areas, the NDVIv and the NDVIm may not have a linear relationship, and it is likely to be a non-linear relationship, so in future research, different machine learning models should be used to correct Fengyun satellite products, such as decision trees, neural networks, and support vector machines. At the same time, we should construct regional machine learning models for parts of China and surrounding areas according to different terrains, different meteorological conditions, and different atmospheric conditions, and use different machine learning methods for different regions to correct Fengyun Satellite products. This may be able to more objectively and accurately correct the NDVIv value to a level equivalent to that of NDVIm.

Secondly, we are currently correcting the Fengyun satellite data from the product level. Although the corrected product is closer to the MODIS product value, it is a physical correction, and the mechanism is not very strong. In the future, we will consider revising the Fengyun Satellite’s NDVI products from the near-infrared and infrared bands, compare and analyze the Fengyun Satellite’s infrared band with the MODIS infrared band, and conduct a comparative analysis on the Fengyun Satellite’s near-infrared band and MODIS’s near-infrared band. It is possible to correct the Fengyun Satellite’s NDVI products to a level closer to that of NDVIm. At the same time, it is also possible to find out the reasons for the inaccuracy of the Fengyun Satellite inversion values, thereby improving the Fengyun Satellite’s own monitoring accuracy.

Thirdly, studies have shown that there is still a certain gap between the NDVIm or NDVIa and the NDVI values observed on the ground^34,35^. Therefore, it is also unreasonable to use the NDVIm as the true value of NDVI. It should also be considered to compare and analyze the Fengyun Satellite’s NDVI product with the NDVI value retrieved from ground observation data, so as to improve the accuracy of the Fengyun Satellite’s NDVI product, and it is also a meaningful thing to improve the accuracy of the Fengyun Satellite.

The product of Fengyun Satellite is not well applied in the process of use at present, and one of the most important reasons is that its observation value differs greatly from the actual value. The NDVI observed by MODIS at present is internationally recognized as a relatively accurate observation value. Therefore, one of the main contents of this study is to build a model, to calibrate the NDVIv to the level of the NDVIm. This goal is realized in this study. In addition, this study aims to form a set of long-time series NDVI observed by MODIS, AVHRR and VIRR at the same time, so that other instruments can replace the missing data of one instrument. Through this study, it is realized that the NDVI value of VIRR instrument of China's Fengyun Satellite can be applied after correction when MODIS or AVHRR data is missing.

## Data Availability

The datasets generated and/or analysed during the current study are available from the corresponding author on reasonable request.

## References

[1] Yang J, Dong C, Lu N (2009). FY-3A: The new generation polar-orbiting meteorological satellite of China. Acta Meteorol. Sin..

[2] Wang J, Guo N (2008). Comparisons of Terra-and Aqua MODIS in band reflectance and vegetation index. Chin. J. Ecol..

[3] Kiage LM, Nan DW (2009). Using NDVI from MODIS to monitor duckweed bloom in Lake Maracaibo, Venezuela. Water Resour. Manag..

[4] Lahet F, Stramski D (2010). MODIS imagery of turbid plumes in San Diego coastal waters during rainstorm events. Remote Sens. Environ..

[5] Kidwell, D. Method for detecting amine-containing drugs in body fluids by sims. US4902627 A[P]. (1990).

[6] Anyamba A, Tucker CJ (2005). Analysis of Sahelian vegetation dynamics using NOAA-AVHRR NDVI data from 1981–2003. J. Arid Environ..

[7] Salim HA, Chen X, Gong J (2007). Analysis of Sudan vegetation dynamics-using NOAA-AVHRR NDVI data from 1993–2003. Asian J. Earth Sci..

[8] Pinzon JE, Tucker CJ (2014). A non-stationary 1981–2012 AVHRR NDVI3g time series. Remote Sens..

[9] Bounoua L, Collatz GJ, Los SO (2000). Sensitivity of climate to changes in NDVI. J. Clim..

[10] Valor E, Caselles V (1996). Mapping land surface emissivity from NDVI: Application to European, African, and South American areas. Remote Sens. Environ..

[11] Chen J (2004). A simple method for reconstructing a high-quality NDVI time-series data set based on the Savitzky–Golay filter. Remote Sens. Environ..

[12] Duarte, L., Teodoro, A. C., Gonçalves, H. Deriving phenological metrics from NDVI through an open source tool developed in QGIS. 924511-924511-9 (2014).

[13] Yoshioka H, Miura T, Huete AR (2000). Analysis of vegetation isolines in red-NIR reflectance space. Remote Sens. Environ..

[14] Goward SN, Markham B, Dye DG (1991). Normalized difference vegetation index measurements from the advanced very high resolution radiometer. Remote Sens. Environ..

[15] ]Gu, Y., Brown, J. F., Verdin, J. P., *et al*. A five-year analysis of MODIS NDVI and NDWI for grassland drought assessment over the central Great Plains of the United States. *Geophys. Res. Lett.* **34**(6) (2007).

[16] Gitelson AA, Kaufman YJ (1998). MODIS NDVI optimization to fit the AVHRR data series—Spectral considerations. Remote Sens. Environ..

[17] Rui F, Ji R, Wu J (2010). Analysis on difference between FY3/MERSI-NDVI and EOS/MODIS-NDVI. Chin. Agric. Sci. Bull..

[18] Yuan Z, Yang A, Zhong B (2015). Cross comparison of the vegetation indexes between Landsat TM and HJ CCD. Remote Sens. Land Resour..

[19] Han-Qiu XU (2011). Cross comparison of ASTER and landsat ETM+ multispectral measurements for NDVI and SAVI vegetation indices. Spectrosc. Spectr. Anal..

[20] Wu W, Yang P (2009). Comparison of two fitting methods of NDVI time series datasets. Trans. Chin. Soc. Agric. Eng..

[21] Jing, L. I., Tai, W. F., Qin, Y. P., *et al*. Analysis of the fitting algorithm of NDVI time series data. *China Mining Mag*.

[22] Sha S, Guo N, Yaohui LI (2013). Contrast of the long-term NDVI/MODIS, NDVI/GIMMS and NDVI/NSMC: A case of Maqu. J. Arid Meteorol..

[23] Kubat, M. *Machine Learning*. (Kluwer Academic Publishers, 2015).

[24] Wang, Y., Xu, R., Liu, B., *et al*. Machine learning and cybernetics.* Commun. Comput. Inf. Sci.* **481** (2014).

[25] Hitouri S, Varasano A, Mohajane M (2022). Hybrid machine learning approach for gully erosion mapping susceptibility at a watershed scale. ISPRS Int. J. Geo Inf..

[26] Pereira F, Mitchell T, Botvinick M (2009). Machine learning classifiers and fMRI: A tutorial overview. Neuroimage.

[27] Staa B, Rn C, Sm C (2021). Prediction of grape yields from time-series vegetation indices using satellite remote sensing and a machine-learning approach. Remote Sens. Appl. Soc. Environ..

[28] Hill P, Temimi M, Kumar A (2020). HABNet: Machine learning, remote sensing based detection of harmful algal blooms. IEEE J. Sel. Top. Appl. Earth Observ. Remote Sens..

[29] Xu T, Guo Z, Xia Y (2019). Evaluation of twelve evapotranspiration products from machine learning, remote sensing and land surface models over conterminous United States. J. Hydrol..

[30] Lary DJ, Alavi AH, Gandomi AH (2016). Machine learning in geosciences and remote sensing. Geosci. Front..

[31] Zhenshan W, Fengzhu Y, Jiangeng W (2022). Consistency of FY-3C VIRR and Terra MODIS NDVI products in Hunan-Jiangxi Region. Sci. Technol. Eng..

[32] Ge M, Zhao J, Bo Z (2017). Comparison of the vegetation indexes between FY-3/VIRR, FY-3/MERSI and EOS/MODIS data. Remote Sens. Technol. Appl..

[33] Munson, M. A. *Outside the Machine Learning Blackbox: Supporting Analysts Before and After the Learning Algorithm*. (Cornell University, 2010).

[34] Fensholt R, Proud SR (2012). Evaluation of earth observation based global long term vegetation trends—Comparing GIMMS and MODIS global NDVI time series. Remote Sens. Environ..

[35] Maselli F, Papale D, Chiesi M (2014). Operational monitoring of daily evapotranspiration by the combination of MODIS NDVI and ground meteorological data: Application and evaluation in Central Italy. Remote Sens. Environ..

